# Hypothermic oxygenated perfusion in liver transplantation: a meta-analysis of randomized controlled trials and matched studies

**DOI:** 10.1097/JS9.0000000000000784

**Published:** 2023-09-21

**Authors:** Gang Tang, Linyu Zhang, Lingying Xia, Jie Zhang, Zhengqiang Wei, Rongxing Zhou

**Affiliations:** a Biliary Surgical Department of West China Hospital; b Analytical and Testing Center; c Center for Translational Medicine, West China Second University Hospital, Sichuan University, Chengdu, Sichuan; d Department of Gastrointestinal Surgery, The First Affiliated Hospital of Chongqing Medical University, Chongqing, People’s Republic of China

**Keywords:** graft preservation, hypothermic oxygenated machine perfusion, liver transplant, liver transplantation, meta-analysis

## Abstract

**Background:**

Hypothermic oxygenated machine perfusion (HOPE) is a novel organ-preservation technology designed to optimize organ quality. However, the effects of HOPE on morbidity and mortality after liver transplantation remain unclear. This meta-analysis evaluated the potential benefits of HOPE in liver transplantation.

**Materials and methods:**

The Embase, Web of Science, PubMed, Cochrane Library, and Scopus databases were searched for articles published up to 15 June 2023 (updated on 12 August 2023). Mean differences (MDs), risk ratios (RRs), and 95% confidence intervals were calculated.

**Results:**

Eleven studies encompassing five randomized controlled trials and six matched studies were included, with a total of 1000 patients. HOPE did not reduce the incidence of major postoperative complications (RR 0.80), primary non-function (PNF) (RR 0.54), reperfusion syndrome (RR 0.92), hepatic artery thrombosis (RR 0.92), renal replacement therapy (RR 0.98), length of hospital stay (MD, −1.38 days), 1-year recipient death (RR 0.67), or intensive care unit stay (MD, 0.19 days) after liver transplantation. HOPE reduced the incidence of biliary complications (RR 0.74), non-anastomotic biliary strictures (NAS) (RR 0.34), early allograft dysfunction (EAD) (RR 0.54), and acute rejection (RR 0.54). In addition, HOPE improved the retransplantation (RR 0.42) and 1-year graft loss rates (RR 0.38).

**Conclusions:**

Compared with static cold storage (SCS), HOPE can reduce the incidence of biliary complications, NAS, EAD, and acute rejection and retransplantation rate after liver transplantation and improve the 1-year graft loss rate. These findings suggest that HOPE, when compared to SCS, can contribute to minimizing complications and enhancing graft survival in liver transplantation. Further research is needed to investigate long-term outcomes and confirm the promising advantages of HOPE in liver transplantation settings.

## Introduction

Highlights

- Hypothermic oxygenated machine perfusion (HOPE) in liver transplantation did not affect primary non-function, reperfusion syndrome, hepatic artery thrombosis, major complications, renal failure, hospital and intensive care unit (ICU) stay, or patient survival.
- HOPE reduced postoperative biliary complications, non-anastomotic biliary strictures, early allograft dysfunction, and acute rejection.
- HOPE improved retransplantation rates and 1-year graft loss, indicating potential benefits for liver transplantation outcomes.

Chronic liver disease has become a major health problem worldwide, causing ~2 million deaths each year^1^. Liver transplantation is the only curative treatment for end-stage liver disease^2^. The 1-year survival rate of patients with end-stage liver disease who undergo liver transplantation can reach 94.2%^3^. Unfortunately, the shortage of donor organs limits the application of liver transplantation. To increase the number of donors, extended standard donors and living donors have been increasingly used^3,4^. However, these donor livers are vulnerable to injury during storage or transplantation, resulting in an increased risk of postoperative biliary complications, early allograft dysfunction (EAD), and primary non-function (PNF)^2,4,5^. Moreover, these additional donor livers were associated with worse long-term survival, thereby leading to further complexities^4^. The current gold standard method for donor liver preservation is static cold storage (SCS)^6^. However, SCS cannot meet the challenges posed by these additional donor livers. Therefore, it is imperative to develop alternative organ-preservation technologies.

Hypothermic oxygenated machine perfusion (HOPE), a technique that provides continuous liver perfusion with cooled oxygenated perfusion fluid, has been associated with improved graft function^7^. Studies have shown that HOPE can enhance mitochondrial recovery and function and is expected to reduce the incidence of biliary complications after liver transplantation in donors after cardiac death (DCD)^8^. However, evidence from current clinical studies is conflicting. A multicenter randomized controlled trial (RCT) by van Rijn *et al*.^9^ demonstrated that HOPE reduced the risk of non-anastomotic biliary strictures (NAS). However, a recent study^10^ reported that the overall incidence of biliary complications in the HOPE group was not significantly different from that in the SCS group.

There is a lack of systematic reviews and meta-analyses exploring the effectiveness of HOPE in liver transplantation. Moreover, matched studies, such as studies with propensity score matching (PSM), can reduce the differences in baseline characteristics between the experimental and control groups, and PSM studies are almost equivalent to RCTs in evaluating the efficacy of interventions^11^. Therefore, incorporating RCTs and matched studies will aid in solving the contradiction of the current research results and provide high-quality evidence. Therefore, by conducting a comprehensive collection of current evidence and meta-analysis of data from both RCTs and matched studies, we aimed to assess the impact of HOPE on postoperative complications and mortality after liver transplantation.

## Methods

### Search strategy

This meta-analysis was conducted according to the Preferred Reporting Items for Systematic Reviews and Meta-Analyses (PRISMA)^12^ (Supplementary Methods 1, Supplemental Digital Content 1, http://links.lww.com/JS9/B64, Supplemental Digital Content 3, http://links.lww.com/JS9/B66) and A MeaSurement Tool to Assess systematic Reviews 2 (AMSTAR2)^13^ (Supplementary Methods 2, Supplemental Digital Content 2, http://links.lww.com/JS9/B65). The protocol was registered in PROSPERO.

Two authors independently conducted a systematic search of the Embase, Web of Science, PubMed, Cochrane Library, and Scopus databases for studies published from inception to 15 June 2023 (updated on 12 August 2023), using the following search strings: (hypothermic oxygenated liver perfusion OR hypothermic oxygenated machine perfusion OR hypothermic oxygenated perfusion OR HOPE) AND (liver grafting OR hepatic grafting OR liver transplant OR liver transplantation OR liver transplantations OR hepatic transplant OR hepatic transplantation OR hepatic transplantations) (Table 1). Language restrictions were not imposed. A list of references for relevant reviews and included studies was also searched.

**Table 1:** Electronic search strategy.

Database	Search term	Number
PubMed (All fields)	#1: hypothermic oxygenated liver perfusion OR hypothermic oxygenated machine perfusion OR hypothermic oxygenated perfusion OR HOPE	#1: 97900
	#2: liver grafting OR hepatic grafting OR liver transplant OR liver transplantation OR liver transplantations OR hepatic transplant OR hepatic transplantation OR hepatic transplantations	#2: 146046
	#3: #1 AND #2	#3: 1036
Embase (All fields)	#1: hypothermic oxygenated liver perfusion OR hypothermic oxygenated machine perfusion OR hypothermic oxygenated perfusion OR HOPE	#1: 158084
	#2: liver grafting OR hepatic grafting OR liver transplant OR liver transplantation OR liver transplantations OR hepatic transplant OR hepatic transplantation OR hepatic transplantations	#2: 253469
	#3: #1 AND #2	#3: 1956
Cochrane Library (All fields)	#1: hypothermic oxygenated liver perfusion OR hypothermic oxygenated machine perfusion OR hypothermic oxygenated perfusion OR HOPE	#1: 7726
	#2: liver grafting OR hepatic grafting OR liver transplant OR liver transplantation OR liver transplantations OR hepatic transplant OR hepatic transplantation OR hepatic transplantations	#2: 8249
	#3: #1 AND #2	#3: 98
Scopus (All fields)	#1: hypothermic oxygenated liver perfusion OR hypothermic oxygenated machine perfusion OR hypothermic oxygenated perfusion OR HOPE	#1: 1388
	#2: liver grafting OR hepatic grafting OR liver transplant OR liver transplantation OR liver transplantations OR hepatic transplant OR hepatic transplantation OR hepatic transplantations	#2: 231955
	#3: #1 AND #2	#3: 837
Web of Science (All fields)	#1: hypothermic oxygenated liver perfusion OR hypothermic oxygenated machine perfusion OR hypothermic oxygenated perfusion OR HOPE	#1: 228539
	#2: liver grafting OR hepatic grafting OR liver transplant OR liver transplantation OR liver transplantations OR hepatic transplant OR hepatic transplantation OR hepatic transplantations	#2: 176243
	#3: #1 AND #2	#3: 1248

### Study selection

The inclusion criteria were: (a) RCTs or matched studies, (b) interventions involving HOPE, (c) comparisons with SCS, (d) evaluation of patients undergoing liver transplantation, and (e) studies evaluating any of the following outcomes: major complications (Clavien–Dindo classification ≥3), biliary complications, hepatic artery thrombosis (HAT), NAS, PNF, EAD, acute rejection, post-reperfusion syndrome, recipient death and graft loss outcomes 1-year post-transplantation, hospital stay, intensive care unit (ICU) stay, retransplantation, costs, and renal replacement therapy.

To minimize the effect of baseline confounders on the results, studies that did not employ matching methods to balance preoperative confounding variables with the HOPE and SCS groups were excluded. Studies with discordant donor types between the HOPE and SCS groups were also excluded. In addition, reviews, letters, abstracts, case reports, non-human studies, and studies without a control group were excluded. Studies evaluating sequential HOPE and other machine-perfusion preservation (e.g. sequential HOPE and normothermic perfusion preservation) in the experimental group were also excluded. This is because the primary objective of this study was to evaluate the impact of HOPE on liver transplant outcomes compared to that of the gold standard of organ-preservation strategies (SCS). Sequential HOPE and other machine-perfusion preservation may affect the true effect of HOPE on liver transplantation due to the combination of other machine perfusion strategies.

### Data extraction

Data, including first author, year, country, study design, sample size, outcomes, donor type, age, recipient MELD (model for end-stage liver disease) scores, perfusion device, and duration of HOPE, cold ischemic time, and warm ischemic time, were extracted from each study by two authors. In cases where relevant data were not available in the published literature, the corresponding author of the study was contacted to obtain the relevant information.

### Quality assessment

The methodological quality of the RCTs was assessed using the Cochrane Collaboration’s risk-of-bias tool 2^14^: (1) randomization process, (2) deviations from intended interventions, (3) missing outcome data, (4) measurement of the outcome, (5) selection of the reported results, and (6) overall risk of bias. The Newcastle–Ottawa Scale (NOS) was used to evaluate the quality of non-RCTs. The total score was 9, and a score ≥6 was considered to be high quality. Two authors independently performed literature retrieval, study selection, data extraction, and quality assessment. Inconsistencies between the two authors were discussed and resolved by a third author.

### Statistical analysis

Risk ratio (RR) with a 95% confidence interval (CI) was calculated for qualitative variables, and mean difference (MD) for quantitative outcomes. Heterogeneity between the studies was assessed using *I*^2^ statistics. *I*^2^≤50% was considered low heterogeneity, and a fixed-effects model was used; *I*^2^>50% was considered high heterogeneity, and a random-effects model was used^15^. To explore the robustness of the results, we used a one-study exclusion test to examine the impact of each study on the pooled effect size. Subgroup analysis was performed based on study type and donor type. The analysis was conducted using Review 5.3. Statistical significance was set at *P*<0.05.

## Results

### Literature search

**Figure 1:**
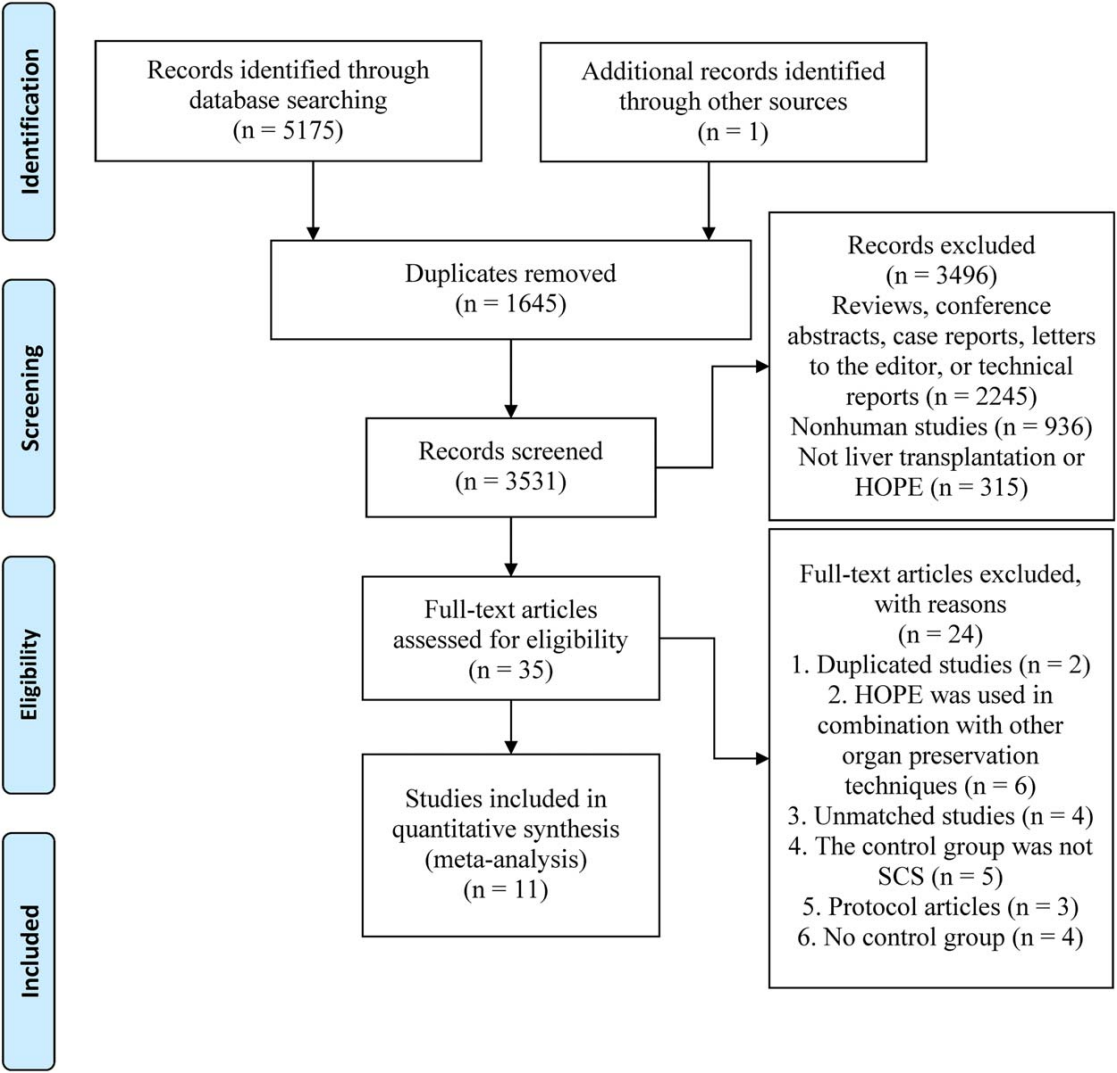
PRISMA flow diagram illustrating the literature retrieval process. HOPE, hypothermic oxygenated machine perfusion; SCS, static cold storage.

A total of 5176 relevant articles were identified. Among them, 1645 duplicate articles were excluded. The examination of the titles and abstracts resulted in the exclusion of 3496 articles that did not meet the inclusion criteria. The remaining 35 articles were evaluated at the full-text level, resulting in the inclusion of 11 studies^2,9,10,16–23^ in the meta-analysis (Fig. 1). The four excluded unmatched studies^24–27^ (three^24–26^ did not use the matching method and one^27^ used inverse probability of treatment weighting, but no post-inverse probability of treatment weighting data were available; even after contacting the corresponding author of the study, no data were available, so this study was classified as a unmatched study) are summarized in Supplementary Table S1 (Supplemental Digital Content 4, http://links.lww.com/JS9/B67).

### Study characteristics

The main characteristics of the 11 included studies are summarized in Table 2. The studies were published between 2015 and 2023 and included 1000 participants (412 in the HOPE group and 588 in the SCS group). Five studies^2,9,10,22,23^ were RCTs, and the remaining six^16–21^ used the matched method. The number of participants in each study ranged from 30 to 170. Three studies^10,16,18^ were conducted in Switzerland, three^19,20,22^ in Italy, two^9,17^ in The Netherlands, and three in Germany^2^, Poland^23^, and France^21^.

**Table 2:** Characteristics of 11 trials included in the meta-analysis.

References	Country	Study design	Sample size	Donor type	Donor age	Recipient age	Recipient MELD scores	Perfusion device	Length of HOPE hours	CIT hours	WIT minutes	Outcomes	NOS
Dutkowski^16^	Switzerland	Matched, PCS	H: 25 S: 50	DCD	H: 54 (36–63) S: 48 (33–51)	H: 60 (57–64) S: 56 (49–59)	H: 13 (9–15) S: 16 (10–21)	ECOPS device (Organ Assist)	2.0 (1.7–2.5)	H: 3.1 (2.4–4.4) S: 6.6 (5.8–7.5)	H: 36 (31–40) S: 33 (27–40)	Hospital stay, ICU stay, NAS, PNF, HAT, EAD, Acute rejection, Total biliary complication, 1-year graft survival, Retransplantation, Renal replacement therapy	7/9
van Rijn^17^	The Netherlands	Matched, PCS	H: 10 S: 20	DCD	H: 53 (47–57) S: 53 (47–58)	H: 57 (54–62) S: 52 (42–60)	H: 16 (15–22) S: 20 (13–24)	Liver Assist device (Organ Assist, Groningen, The Netherlands)	2.1 (2.1–2.3)	H: 6.0 (5.2–6.6) S: 7.1 (6.7–8.1)	H: 26 (23–42) S: 33 (29–41)	Hospital stay, ICU stay, PNF, NAS, HAT, EAD, Total biliary complication, 1-year graft survival, 1-year patient survival, Retransplantation, Renal replacement therapy	9/9
Schlegel^18^	Switzerland	Matched, RCS	H: 50 S: 50	DCD	H: 57 (47–67) S: 53 (33–60)	H: 58 (56–62) S: 57 (51–61)	H: 11 (8–14) S: 11.8 (8.5–15.8)	Liver Assist device (Organ Assist)	2 (1.6–2.4)	H: 4.4 (3.5–5.2) S:4.7 (4.3–5.3)	H: 36 (31–40) S: 25.5 (21–31)	PNF, NAS, Total biliary complication, Acute rejection, Renal replacement therapy	7/9
Patrono^19^	Italy	RCS, PSM	H: 25 S: 50	DBD	H: 74.3 (10.9) S: 74.9 (10.3)	H: 56.3 (9) S: 55.9 (7.4)	H: 15.3 (8.6) S: 15.5 (8.5)	Liver Assist device (Organ Assist, Groningen, The Netherlands)	3.1 (0.8)	H: 5.2 (0.9) S:6.5 (1.2)	H: 24 (5) S: 23 (7)	Hospital stay, ICU stay, Acute rejection, NAS, EAD, PNF, Total biliary complication, Renal replacement therapy, Major complications	8/9
Ravaioli^20^	Italy	Matched, PCS	H: 10 S: 30	DBD	H: 77.5 (60–84) S: 75.5 (53–85)	H: 57.5 (50–68) S: 60.5 (48–68)	H: 13 (7–16) S: 13.5 (7–20)	Developed by Medica S.P.A and Centro Iperbarico S.R.L. under the scientific management of the author	2.2 (1–3.5)	H: 7.1 (6.1–9.6) S:7 (5.4–10)	NA	PNF, EAD, Hospital stay, Acute rejection, 1-year graft survival, 1-year patient survival, Total biliary complication, Major complications	8/9
Czigany^2^	Germany	Multicenter, RCT	H: 23 S: 23	DBD	H: 73 (60–78) S: 71 (59–78)	H: 60 (52–64) S: 63 (56–67)	H: 13 (9–18) S: 17 (8–25)	Liver Assist	2.4 (1.7–3.4)	H: 6.3 (5.2–7.8) S:8.4 (7.8–9.7)	H: 39 (35–54) S: 45 (39–52)	Hospital stay, ICU stay, Acute rejection, EAD, PNF, HAT, Cost, Total biliary complication, 1-year graft survival, 1-year patient survival, Retransplantation, Major complications, Renal replacement therapy	–
Rayar^21^	France	PCS, PSM	H: 25 S: 69	DBD	H: 70 (45–87) S: 72 (25–88)	H: 63 (43–69) S: 62 (36–70)	H: 18.3 (7–37) S: 18.3 (5–40)	Liver Assist	2 (1.3–4.2)	H: 8.8 (6.3–13.7) S:9.3 (3.5–12.0)	NA	PNF, NAS, EAD, Hospital stay, ICU stay, Total biliary complication, 1-year graft survival, 1-year patient survival, Cost, Major complications, Retransplantation, Renal replacement therapy	8/9
van Rijn^9^	The Netherlands	Multicenter, RCT	H: 78 S: 78	DCD	H: 52 (43–57) S: 49 (37–59)	H: 60 (52–65) S: 60 (52–65)	H: 14 (10–19) S: 16 (10–22)	Liver Assist device (Organ Assist)	2.2 (2.0–2.6)	H: 6.2 (5.3–6.9) S:6.8 (5.9–8.0)	H: 29 (22–33) S: 27 (21–35)	Hospital stay, ICU stay, EAD, PNF, Retransplantation, NAS, Total biliary complication, Major complications, Total, Post-reperfusion syndrome, HAT, Renal replacement therapy	–
Ravaioli^22^	Italy	RCT	H: 55 S: 55	DBD	H: 76 (64–81) S: 72 (59–77)	H: 57 (47–65) S: 60 (53–66)	H: 15 (10–18) S: 14 (9–20)	Vitasmart (Bridge to Life, DG, USA) machine	2.4 (2-3.1)	H: 4.3 (3.6–5.4) S:7.0 (6.0–7.5)	NA	Hospital stay, ICU stay, EAD, PNF, HAT, Retransplantation, Post-reperfusion syndrome	–
Grąt^23^	Poland	RCT	H: 26 S: 78	DBD	H: 53 (40–60) S: 44 (35–56)	H: 46 (39–62) S: 51 (41–60)	H: 12 (8–21) S: 14 (10–21)	Liver Assist device (Organ Assist, now XVIVO)	2.0	H: 7.5 (7.0–9.2) S:9.5 (8.0–10.5)	NA	ICU stay, EAD, PNF, Major complications, Total biliary complication, Post-reperfusion syndrome	–
Schlegel^10^	Switzerland	Multicenter, RCT	H: 85 S: 85	DBD	H: 59 (48–72) S: 62 (44–71)	H: 60 (51–64) S: 57 (49–64)	H: 20 (11–28) S: 19 (12–26)	Liver Assist	1.6 (1.2–2.3)	H: 6.2 (5.0–7.9) S:7.1 (5.9–8.1)	NA	Hospital stay, ICU stay, EAD, NAS, Total biliary complication, 1-year graft survival, 1-year patient survival, Major complications, HAT	–

CIT, cold ischemic time; DBD, donors after brain death; DCD, donors after cardiac death; EAD, early allograft dysfunction; HAT, hepatic artery thrombosis; ICU, intensive care unit; MELD, model for end-stage liver disease; NAS, non-anastomotic biliary stricture; PCS, prospective cohort study; PNF, primary non-function; PSM, propensity score matching; RCS, retrospective cohort study; RCT, randomized controlled trial; WIT, warm ischemic time.

### Methodological quality

**Figure 2:**
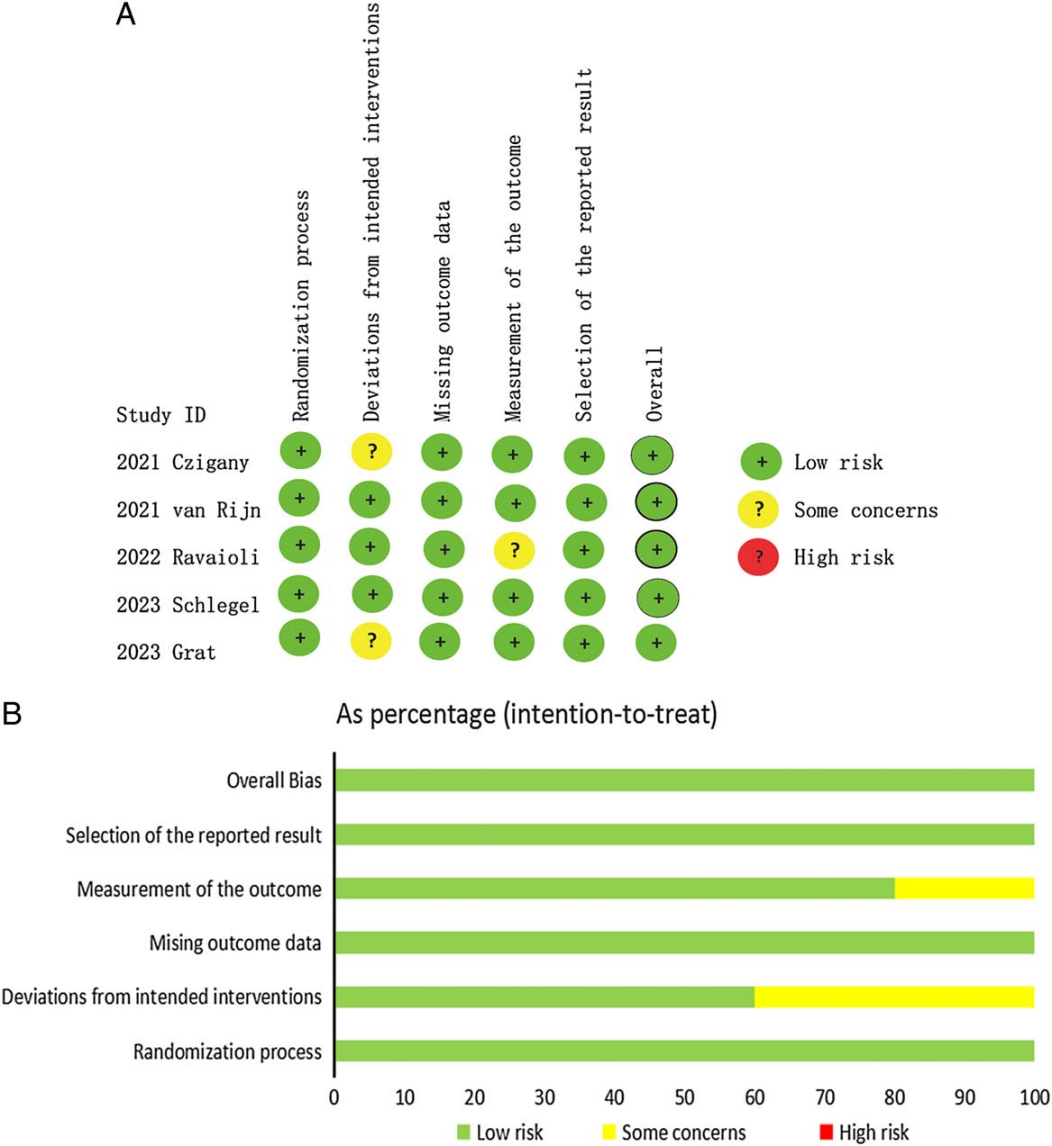
Risk of bias for RCTs. (A) Risk of bias summary. (B) Risk of bias graph.

All included non-RCT studies were of good quality, with scores of six or more (Table 2). All five RCTs^2,9,10,22,23^ were considered to have a low risk of bias. The results of the risk of bias for each study are shown in Figure 2.

### Meta-analysis

#### Total biliary complications

**Figure 3:**
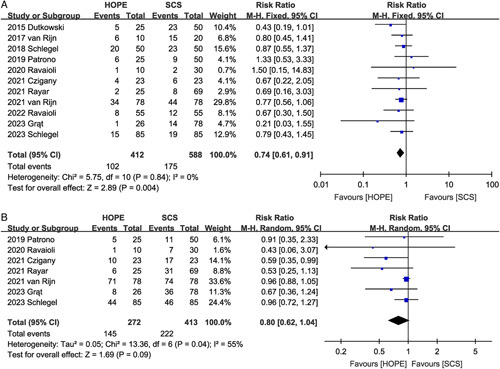
Forest plot of (A) biliary complications and (B) major complications between HOPE and SCS. CI, confidence interval; HOPE, hypothermic oxygenated machine perfusion; SCS, static cold storage.

Eleven datasets^2,9,10,16–23^ were used to evaluate the effects of HOPE on the total postoperative biliary complications. Compared to the control group, HOPE effectively reduced the risk of postoperative total biliary complications (RR 0.74, 95% CI 0.61, 0.91, *P*=0.004) (Fig. 3A) (Table 3). However, in the subgroup analysis, the decrease in total biliary complications was significant only in the DCD group (RR 0.74, 95% CI 0.59, 0.94, *P*=0.01) (Table 4).

**Table 3:** Summary of results from all outcomes.

Indicators	Number of studies	Events for HOPE	Events for SCS	Effect size	95% CI
Major complications	7	145/272	222/413	0.8	0.62, 1.04
Total biliary complications	11	102/412	175/588	0.74	0.61, 0.91
Hepatic artery thrombosis	6	6/276	9/311	0.92	0.36, 2.34
Non-anastomotic biliary stricture	7	13/298	50/402	0.34	0.19, 0.61
Primary non-function	10	3/327	16/503	0.54	0.22, 1.30
Early allograft dysfunction	10	71/362	196/538	0.54	0.42, 0.68
Acute rejection	5	14/133	38/203	0.54	0.31, 0.94
Post-reperfusion syndrome	3	56/159	74/211	0.92	0.57, 1.48
Retransplantation rates	5	6/266	19/310	0.42	0.19, 0.93
Renal replacement therapy rates	7	30/236	38/340	0.98	0.64, 1.51
One-year graft loss	7	12/233	53/332	0.38	0.21, 0.68
One-year recipient death	5	8/153	20/227	0.67	0.31, 1.45
Hospital stay	9	–	–	−1.38	–2.87, 0.12
Intensive care unit stay	9	–	–	0.19	−0.19, 0.56
Hospitalization costs	2	–	–	−9.43	−35.57, 16.70

**Table 4:** Summary of results from all subgroup analyses.

Indicators	Subgrouped by	The number of studies	Effect size	95% CI	*I*^2^ (%)	*P* for between subgroup heterogeneity
Major complications	Types of study	–	–	–	–	0.33
	RCT	4	0.86	0.68, 1.09	56	–
	MS	3	0.63	0.36, 1.11	0	–
Total biliary complications	Types of study	–	–	–	–	0.61
	RCT	5	0.71	0.54, 0.93	0	–
	MS	6	0.79	0.58, 1.08	0	–
	Donor type	–	–	–	–	0.99
	DCD	4	0.74	0.59, 0.94	0	–
	DBD	7	0.74	0.51, 1.08	0	–
HAT	Types of study	–	–	–	–	0.46
	RCT	4	1.18	0.38, 3.63	0	–
	MS	2	0.54	0.09, 3.10	0	–
	Donor type	–	–	–	–	0.53
	DCD	3	0.70	0.19, 2.53	0	–
	DBD	3	1.29	0.32, 5.13	21	–
NAS	Types of study	–	–	–	–	0.92
	RCT	2	0.35	0.14, 0.86	0	–
	MS	5	0.33	0.15, 0.72	0	–
	Donor type	–	–	–	–	0.28
	DCD	4	0.29	0.15, 0.57	0	–
	DBD	3	0.65	0.18, 2.32	0	–
PNF	Types of study	–	–	–	–	0.59
	RCT	4	0.40	0.10, 1.68	0	–
	MS	6	0.67	0.22, 2.06	3	–
	Donor type	–	–	–	–	0.30
	DCD	4	0.26	0.05, 1.50	0	–
	DBD	6	0.77	0.27, 2.21	0	–
EAD	Types of study	–	–	–	–	0.41
	RCT	5	0.50	0.38, 0.66	16	–
	MS	5	0.62	0.41, 0.93	0	–
	Donor type	–	–	–	–	0.67
	DCD	3	0.58	0.38, 0.87	0	–
	DBD	7	0.52	0.39, 0.69	23	–
Acute rejection	Donor type	–	–	–	–	0.07
	DCD	2	0.31	0.13, 0.75	68	–
	DBD	3	0.90	0.43, 1.91	0	–
Patient survival	Types of study	–	–	–	–	0.75
	RCT	2	0.75	0.27, 2.07	0	–
	MS	3	0.58	0.18, 1.93	0	–
Graft survival	Types of study	–	–	–	–	0.92
	RCT	3	0.37	0.16, 0.85	0	–
	MS	4	0.39	0.17, 0.88	0	–
	Donor type	–	–	–	–	0.33
	DCD	2	0.23	0.07, 0.80	0	–
	DBD	5	0.46	0.24, 0.90	0	–
Hospital stay	Types of study	–	–	–	–	0.77
	RCT	4	−1.54	−3.37, 0.30	67	–
	MS	5	−1.06	−3.64, 1.53	0	–
	Donor type	–	–	–	–	0.50
	DCD	3	−1.94	−4.14, 0.27	0	–
	DBD	6	−0.90	−2.94, 1.14	58	–
ICU stay	Types of study	–	–	–	–	0.70
	RCT	5	0.21	−0.19, 0.61	56	–
	MS	4	−0.01	−1.07, 1.05	0	–
	Donor type	–	–	–	–	0.17
	DCD	3	0.55	−0.09, 1.19	0	–
	DBD	6	−0.00	−0.46, 0.46	38	–
Renal replacement therapy	Types of study	–	–	–	–	0.30
	RCT	2	0.75	0.38, 1.47	0	–
	MS	5	1.19	0.68, 2.08	1	–
	Donor type	–	–	–	–	0.30
	DCD	4	1.13	0.68, 1.89	27	–
	DBD	3	0.68	0.31, 1.53	0	–

DBD, donors after brain death; DCD, donors after cardiac death; EAD, early allograft dysfunction; HAT, hepatic artery thrombosis; ICU, intensive care unit; MS, matched study; NAS, non-anastomotic biliary stricture; PNF, primary non-function; RCT, randomized controlled trial.

#### Major complications

Seven studies^2,9,10,19–21,23^, including 685 patients, provided data for major complication analysis, and the pooled results showed that HOPE did not reduce the incidence of major complications (RR 0.80, 95% CI 0.62, 1.04; Heterogeneity: *I*^2^=55%, *P*=0.04) compared with livers preserved with SCS (Fig. 3B).

#### Hepatic artery thrombosis

**Figure 4:**
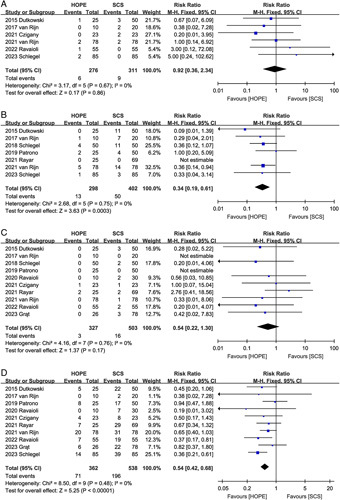
Forest plot of (A) hepatic artery thrombosis, (B) non-anastomotic biliary stricture, (C) primary non-function, and (D) early allograft dysfunction between HOPE and SCS. CI, confidence interval; HOPE, hypothermic oxygenated machine perfusion; SCS, static cold storage.

HAT was reported in six studies^2,9,10,16,17,22^ (four RCTs and two matched studies), showing that HOPE did not reduce the risk of HAT (RR 0.92, 95% CI 0.36, 2.34, *P*=0.86). Notably, no significant heterogeneity was observed among studies (*I*^2^=0%, *P*=0.67) (Fig. 4A).

#### Non-anastomotic biliary strictures

Evidence from seven studies^9,10,16–19,21^ suggested that HOPE was effective in reducing the incidence of NAS (RR 0.34, 95% CI 0.19, 0.61, *P*=0.0003), and there was no significant heterogeneity among the studies (*I*^2^=0%, *P*=0.75) (Fig. 4B). When subgroup analysis was performed according to the study type, both RCT (RR 0.35, 95% CI 0.14, 0.86, *P*=0.02) and non-RCT studies (RR 0.33, 95% CI 0.15, 0.72, *P*=0.005) showed that HOPE was effective in reducing the incidence of NAS.

#### Primary non-function

Ten trials^2,9,16–23^ reported PNF data. There was no significant difference in the incidence of postoperative PNF (RR 0.54, 95% CI 0.22, 1.30; Heterogeneity: *I*^2^=0%, *P*=0.76) between the livers receiving HOPE storage and those receiving SCS preservation (Fig. 4C), which was also confirmed by subgroup analysis.

#### Early allograft dysfunction

EAD was described in ten studies^2,9,10,16,17,19–23^, and the total effect size showed that the use of HOPE was associated with a reduced risk of EAD (RR 0.54, 95% CI 0.42, 0.68, *P*<0.00001), with no significant heterogeneity between the studies (*I*^2^=0%, *P*=0.48) (Fig. 4D). Evidence from both RCT (RR 0.50, 95% CI 0.38, 0.66, *P*<0.00001) and non-RCT studies (RR 0.62, 95% CI 0.41, 0.93, *P*=0.02) suggests that HOPE is associated with a reduced incidence of EAD.

#### Acute rejection

**Figure 5:**
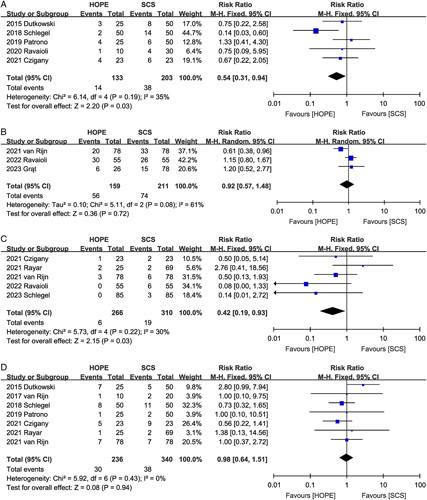
Forest plot of (A) acute rejection, (B) post-reperfusion syndrome, (C) retransplantation, and (D) renal replacement therapy between HOPE and SCS. CI, confidence interval; HOPE, hypothermic oxygenated machine perfusion; SCS, static cold storage.

Five studies^2,16,18–20^ compared acute rejection rates between the HOPE and SCS groups. The pooled results showed that HOPE was effective in reducing the incidence of acute rejection (RR 0.54, 95% CI 0.31, 0.94, *P*=0.03) (Fig. 5A) and that the benefit of HOPE was greater in the DCD subgroup (RR 0.31, 95% CI 0.13, 0.75, *P*=0.009).

#### Post-reperfusion syndrome

Post-reperfusion syndrome was reported in three studies^9,22,23^, and there was no significant difference in the incidence of post-reperfusion syndrome (RR 0.92, 95% CI 0.57, 1.48; Heterogeneity: *I*^2^=61%, *P*=0.08) between the two groups (Fig. 5B).

#### Retransplantation rates

Retransplantation rates were reported in seven studies. Notably, Dutkowski *et al*.^16^ reported that HOPE (0/25) was effective in reducing the rate of retransplantation due to ischemic cholangiopathy or PNF compared to SCS (9/50). Moreover, van Rijn *et al*.^17^ described only the effect of HOPE on the rate of retransplantation due to biliary complications. Therefore, we did not include data from these two studies. The pooled results of the remaining five^2,9,10,21,22^ studies showed that HOPE was effective in reducing the incidence of postoperative retransplantation (RR 0.42, 95% CI 0.19, 0.93, *P*=0.03) (Fig. 5C).

#### Renal replacement therapy rates

Seven studies^2,9,16–19,21^ assessed the effect of HOPE on renal failure, which led to increased renal replacement therapy rates. The results indicate that the HOPE and SCS groups had comparable renal replacement therapy rates (RR 0.98, 95% CI 0.64, 1.51; Heterogeneity: *I*^2^=0%, *P*=0.43) (Fig. 5D).

#### One-year graft loss

**Figure 6:**
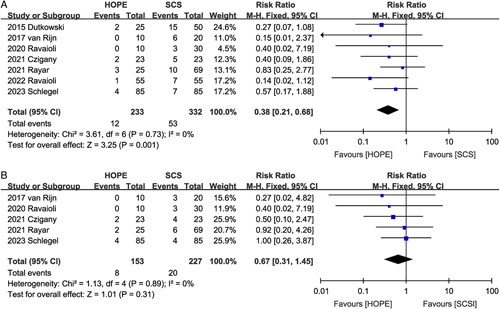
Forest plot of (A) 1-year graft loss and (B) 1-year recipient death between HOPE and SCS. CI, confidence interval; HOPE, hypothermic oxygenated machine perfusion; SCS, static cold storage.

Seven studies^2,10,16,17,20–22^ reported the 1-year graft loss rates. HOPE improved the 1-year graft loss rate (RR 0.38, 95% CI 0.21, 0.68, *P*=0.001) compared to SCS, with no significant heterogeneity (*I*^2^=0%, *P*=0.73) between the studies (Fig. 6A). However, subgroup analysis indicated that improvement in 1-year graft loss was both observed in the DCD group (RR 0.23, 95% CI 0.07, 0.80, *P*=0.02) and in the donors after brain death (DBD) group (RR 0.46, 95% CI 0.24, 0.90, *P*=0.02).

#### One-year recipient death

Pooled data from 380 participants in five studies^2,10,17,20,21^ showed that HOPE did not improve recipient death at the 1-year point (RR 0.67, 95% CI 0.31, 1.45, *P*=0.31), and no significant heterogeneity was observed between studies (*I*^2^=0%, *P*=0.89) (Fig. 6B).

#### Hospital stay

**Figure 7:**
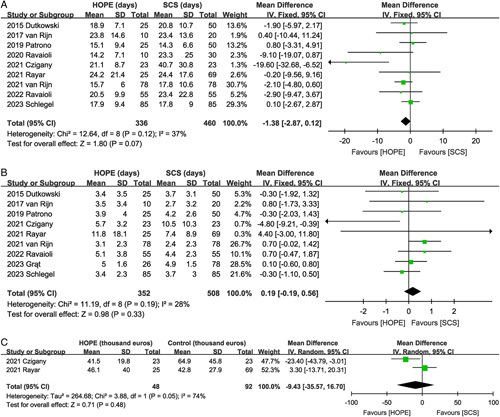
Forest plot of (A) hospital stay, (B) intensive care unit stay, and (C) costs between HOPE and SCS. CI, confidence interval; HOPE, hypothermic oxygenated machine perfusion; SCS, static cold storage.

Nine trials^2,9,10,16,17,19–22^ reported the length of hospital stay. The length of hospital stay was comparable between the HOPE and SCS groups with no significant heterogeneity (MD, −1.38 days; 95% CI, −2.87, 0.12, *P*=0.07; *I*^2^=37%) (Fig. 7A).

#### Intensive care unit stay

Data on ICU stay were reported in nine studies^2,9,10,16,17,19,21–23^. The pooled analysis showed that HOPE did not reduce ICU stay (MD, 0.19 days; 95% CI, −0.19, 0.56, *P*=0.33; *I*^2^=28%) (Fig. 7B).

#### Hospitalization costs

Only two studies described hospitalization costs^2,21^, and the combined total effect size indicated no significant difference in hospitalization costs (MD, −9.43 thousand euros; 95% CI, −35.57, 16.70, *P*=0.48; *I*^2^=74%) between the HOPE and SCS groups (Fig. 7C).

#### Sensitivity analysis

Sensitivity analysis showed that no single study affected the overall effect size of biliary complications, post-reperfusion syndrome, 1-year recipient death, 1-year graft loss, renal replacement therapy rates, HAT, NAS, EAD, ICU stay, and PNF. The size of the pooled effect of the retransplantation rates was influenced by van Rijn *et al*.^9^ (RR 0.38, 95% CI 0.14, 1.02, *P*=0.06), Schlegel *et al*.^10^ (RR 0.48, 95% CI 0.21, 1.11, *P*=0.08), or Ravaioli *et al*.^22^ (RR 0.59, 95% CI 0.25, 1.41, *P*=0.24). The overall effect size for the hospital stay changed when the study by Schlegel *et al*.^10^ (MD, −1.99 days; 95% CI, −3.77, −0.21, *P*=0.03) or the study by Patrono *et al*.^19^ (MD, −1.71 days; 95% CI, −3.32, −0.10, *P*=0.04) was excluded. The size of the pooled effect of the acute rejection rates was influenced by Schlegel *et al*.^18^ (RR 0.86, 95% CI 0.45, 1.62, *P*=0.63). The overall effect size for the major complication changed when the study by van Rijn *et al*.^9^ (RR 0.80, 95% CI 0.64, 0.99, *P*=0.04) was excluded.

## Discussion

HOPE has attracted considerable attention from the transplant community in recent years. Despite the theoretical protective effect of HOPE on mitochondrial function recovery, the application of HOPE in liver transplantation is still limited due to the lack of convincing evidence to prove the clinical value of this approach in liver transplantation^20,28^. Our meta-analysis, based on evidence from high-quality RCTs and matched studies, showed that although HOPE did not reduce the incidence of PNF, reperfusion syndrome, recipient death at the 1-year rate, HAT, renal failure requiring replacement therapy, major postoperative complications, length of hospital stay, or ICU time after liver transplantation, it reduced the incidence of biliary complications, NAS, EAD, and acute rejection. In addition, HOPE improved the retransplantation rates and 1-year graft loss rates.

Previous studies have shown that mitochondria are the initiating factors that trigger an entire inflammatory cascade, leading to cellular damage during liver transplantation^29^. During ischemia, there is a substantial loss of ATP because of the suspension of electron transport through the respiratory chain complex due to hypoxia. When feeding is restored, mitochondria immediately metabolize succinate to produce ATP, resulting in the rapid release of reactive oxygen species. Reactive oxygen species can lead to the damage and death of some cells and promote an inflammatory response, leading to damage of hepatocytes and bile duct cells^29^. Therefore, protection of mitochondria may be the key to improving cellular function and mitigating liver damage.

It has been reported that HOPE alleviated microcirculation disorders and endoplasmic reticulum stress, promoted ATP and glycogen synthesis, improved mitochondrial function, and reduced donor liver injury during rewarming^7,30,31^. Restoration of ATP levels reduces the production of free radical oxygen and damp-related molecular pattern molecules after reperfusion and leads to reduced activation of Kupffer cells and the endothelium, thereby limiting ischemia-reperfusion injury^17^. Notably, van Rijn *et al*.^17^ found an 11-fold increase in intrahepatic ATP levels in the dual HOPE group compared with the SCS group. Kron *et al*.^32^ demonstrated that HOPE significantly reduced hepatocyte oxidative stress, nuclear damage, and liver fibrosis after liver transplantation. Additionally, HOPE reduced liver ischemia-reperfusion injury induced by liver transplantation by inhibiting inflammatory responses^33^. Moreover, Patrono *et al*.^27^ found that HOPE reduced neutrophil infiltration in reperfusion biopsy tissues. Therefore, HOPE may have potential clinical benefits, particularly in terms of complications and functional protection.

Complications after liver transplantation not only increase hospitalization costs but also impair the long-term prognosis of patients^34,35^. Among these complications, biliary complications are the most commonly encountered. NAS is an important factor that leads to graft loss and retransplantation^4,29^. In addition, previous evidence suggests that EAD may be associated with reduced survival after liver transplantation^4^.

Our study has several important clinical implications. Notably, it provides the latest and comprehensive evidence supporting the significant reduction of the incidences of total biliary complications, EAD, and NAS, with the use of HOPE. These benefits were associated with improved graft and patient outcomes, which were also confirmed in our study. Notably, a lower retransplantation rate and 1-year graft loss rate were depicted in the HOPE group compared to the SCS group. However, no difference in the recipient death at the 1-year rate was observed between the two groups in our study, which may be related to the high 1-year survival rate of liver transplant patients. Therefore, further studies are required to confirm the effect of HOPE on long-term patient prognosis. Similarly, a retrospective non-PSM study^26^ of 100 liver transplant recipients showed that HOPE reduced the incidence of EAD by 44%. Patrono *et al*.^27^ found that the use of dual HOPE was associated with a significant reduction in EAD, major complications, and comprehensive complication index. In addition, dual HOPE significantly improved the graft and patient survival rates^27^. A recently published retrospective study found that the sequential administration of Normothermic Regional Perfusion and HOPE was effective in reducing the risk of ischemic cholangiopathy^36^. In addition, Rossignol *et al*.^25^ were the first to explore the use of HOPE in split transplantation; however, this was a phase 1 study with a small sample size and limited statistical power. The 90-day overall complication rate was significantly lower in the HOPE group than in the SCS group (12/24 vs. 7/16); however, the difference was not significant. In our meta-analysis, the incidence of PNF was comparable between the HOPE and SCS groups. This may be due to the low incidence of PNF after liver transplantation (5–8%)^4^. The incidence of PNF in our study was 0.9% (3/327) in the HOPE group and 3.2 (16/503) in the SCS group. An insufficient amount of data may be an important obstacle to drawing beneficial conclusions of HOPE. Therefore, the benefits of HOPE for PNF need to be verified in additional studies with larger sample sizes. HOPE incurs additional costs for perfusion devices, kits, and machine perfusion solutions. Machine perfusion surgery is estimated to incur an additional cost of €5298 per patient^21^. However, our study suggests that the HOPE and SCS groups are comparable in terms of hospitalization costs, which may be related to fewer postoperative complications in the HOPE group.

Acute cellular rejection is a common lymphocyte-mediated immune response that occurs following liver transplantation^37^. Rejection may affect the survival rates after liver transplantation^7^. HOPE has also been shown to reduce the response of the innate immune system after transplantation because of its protective effects on mitochondria and its anti-inflammatory effects^29^. Schlegel *et al*.^38^ found that HOPE reduces T-cell numbers and cytokine levels and regulates the immune system. Our study revealed that HOPE significantly reduced the incidence of acute rejection, which may be related to its effects on the immune system. Similarly, a recent meta-analysis published by Maspero *et al*.^39^, which included eight studies, mostly focusing on HOPE (six), showed that HOPE reduces postoperative acute cellular rejection.

In addition, DCD liver donors are at a higher risk in liver complications than donors with DBD^10^. Studies have reported that the risk of NAS after liver transplantation in donors with DCD is three times higher than that in donors with DBD^40^. Of note, the results of our subgroup analysis indicated that a greater benefit of HOPE was observed in the subgroup of DCD donors, including total biliary complications, acute rejection, NAS, and graft loss. This suggests that HOPE has greater potential as DCD donors.

Previous studies have shown that machine perfusion helps reduce the length of hospital stay^3,41^. Although we found a benefit of HOPE in reducing postoperative biliary complications, HOPE did not reduce the length of postoperative hospital stay. This finding is similar to that of two previous studies^25,27^.

Our study has several strengths. First, we conducted a comprehensive search of multiple databases (Embase, Web of Science, Scopus, PubMed, and Cochrane Library). This comprehensive approach ensured that we captured a wide range of relevant studies. Second, our study included the most recent and important studies, further enhancing the relevance of our findings. Finally, we only included RCTs and matched studies, which contributes to the reliability of our findings.

However, our study also has the following limitations: First, some of the included studies had small samples, which may be limited by the complexity and rarity of the liver transplant itself. Second, there is heterogeneity in graft quality or donors, such as the median age of donors included in the study, which ranges from 48 to 77.5 years. Finally, some outcome measures (post-reperfusion syndrome, retransplantation rate, 1-year recipient death, and cost) were based on evidence from a small number of studies, and may be underpowered. Therefore, it is not possible to determine whether HOPE confers additional benefits, and further research is required to explore the effects of HOPE on these outcome measures.

## Conclusions

This meta-analysis, based on currently available and updated evidence, suggests that although HOPE did not reduce the incidence of major postoperative complications, PNF, reperfusion syndrome, HAT, renal failure requiring replacement therapy, length of hospital stay, and ICU stay after liver transplantation, it reduced the incidence of biliary complications, NAS, EAD, and acute rejection.

In addition, HOPE improved the retransplantation rates and 1-year graft loss rates. Of note, the subgroup analysis indicated that a greater benefit of HOPE was observed in the subgroup of DCD, including total biliary complications, acute rejection, NAS, and graft loss. Further RCTs with long-term follow-ups are needed to confirm the benefits of HOPE in liver transplantation.

## Ethical approval

Ethical approval is not applicable.

## Consent

This meta-analysis was based on former studies and the consent was not required in this meta-analysis.

## Sources of funding

This work was financially supported by National Natural Science Foundation of China (22004088) and Science & Technology Support Project of Sichuan Province (2023YFS0183).

## Author contribution

G.T., L.Y.Z., Z.Q.W., and L.Y.X.: designed the study; G.T. and J.Z.: designed and conducted the search strategy; G.T., L.Y.Z., and J.Z.: screened the studies for eligibility, completed the data extraction, and assessed the risk of bias; G.T., L.Y.Z., and R.X.Z.: analyzed the data; G.T.: wrote the manuscript in consultation with L.Y.Z., Z.Q.W., and L.Y.X. All authors discussed the results and contributed to the final manuscript. J.Z. and R.X.Z. supervised the project. All authors read and approved the final manuscript.

## Conflicts of interest disclosure

The authors declare no relevant conflicts of interest.

## Research registration unique identifying number (UIN)

1. Name of the registry: PROSPERO.
2. Unique identifying number or registration ID: CRD42023435615.
3. Hyperlink to your specific registration (must be publicly accessible and will be checked): https://www.crd.york.ac.uk/PROSPERO/display_record.php?RecordID=435615

## Guarantor

Rongxing Zhou.

## Data availability statement

The raw data were all collected in the included studies. We declare the authenticity of the data. No datasets were generated or analyzed during the current study.

## Provenance and peer review

Not commissioned, externally peer-reviewed.

## Presentation

None.

## Supplementary Material

SUPPLEMENTARY MATERIAL
