# Supplementary figures and images for: Hypothermic oxygenated perfusion in liver transplantation: a meta-analysis of randomized controlled trials and matched studies
Source: Int J Surg. 2023 Sep 21;110(1):464–77. doi: 10.1097/JS9.0000000000000784 (PMC10793758; doi:10.1097/JS9.0000000000000784)

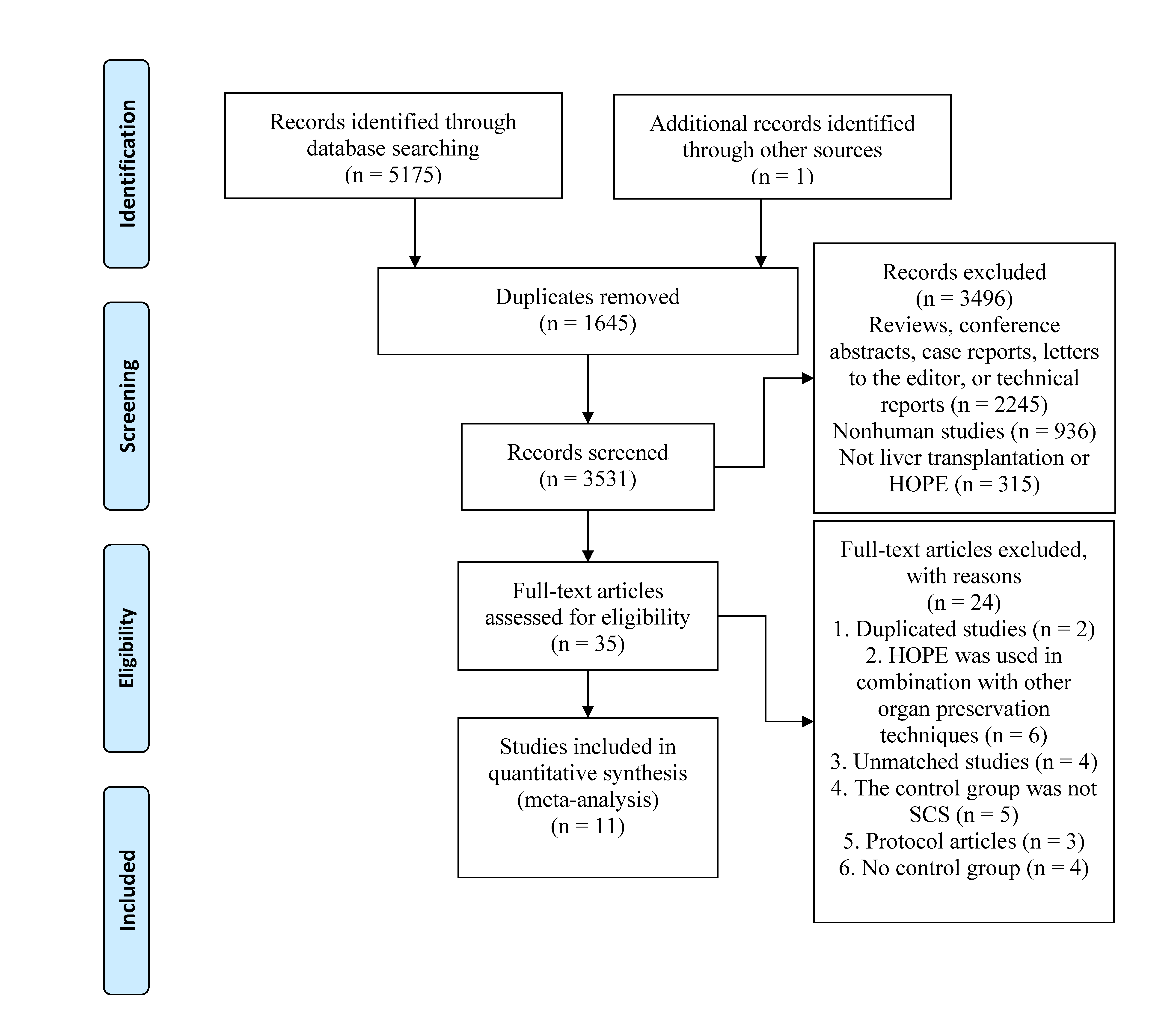

Supplement: SUPPLEMENTARY MATERIAL [file js9-110-464-s003.tiff]
