# Supplementary material for: Hypothermic oxygenated perfusion in liver transplantation: a meta-analysis of randomized controlled trials and matched studies
Source: Int J Surg. 2023 Sep 21;110(1):464–77. doi: 10.1097/JS9.0000000000000784 (PMC10793758; doi:10.1097/JS9.0000000000000784)
Supplement: SUPPLEMENTARY MATERIAL [file js9-110-464-s004.doc]

**Supplementary Table S1. Characteristics of unmatched trials.**

| First author, year | Country | Study design | Sample size | Donor age | Recipient age | Recipient MELD scores | Perfusion Device | Length of  HOPE hours | CIT hours | WIT min | Outcomes |
| --- | --- | --- | --- | --- | --- | --- | --- | --- | --- | --- | --- |
| Mueller, 2020 | Switzerland | RCS | H: 70  S: 70 | H: 59.5(48.75-72.0)  S: 52(35-66.25) | H: 60.5(56.75-66.25)  S: 61.0(57.0-66.0) | H: 10(8-14)  S: 7(5-10) | ECOPS device (Organ  Assist) | 2.0(1.7-2.5) | H: 4.05(3.2-5)  S: 6.856(5.87-7.6) | H: 30.5(26-35.0)  S: 19(14.25-23) | HOPE reduced the rate of tumor recurrence |
| Rossignol, 2022 | France | PCS | H-Adult: 8  S-Adult: 12  H- Pediatric: 8  S- Pediatric: 12 | H: 21(19-27)  S: 20(17-28) | H-Adult: 58(43-60)  S-Adult: 47.5(26-63.5)  H- Pediatric: 42.1(23.6-65)  S- Pediatric: 24.8(11.6-39.5) | H-Adult: 12(8-18)  S-Adult: 9(8-13)  H- Pediatric: 17(13-21)  S- Pediatric: 24(16-39) | Liver Assist Machine perfusion device (XVIVO,  Groningen, The Netherlands) | H-Adult: 2.6(2.1-2.8)  H- Pediatric: | H-Adult: 7.2(6.3-8.5)  S-Adult: 9.1(7.5-10)  H- Pediatric: 8.2(7.8-8.6)  S- Pediatric: 9.1(8.6-9.6) | NA | No early graft loss occurred and liver graft–related adverse events were comparable between SCS and HOPE. HOPE did not increase split procedure duration but resulted in reduced SCS duration leading to reduced ischemia/reperfusion injury on reperfusion biopsies |
| Horné, 2022 | Germany | RCS | H: 50  S: 50 | H: 49.2(44.4-54.0)  S: 55.1(50.5-59.6) | H: 53.0(53.0-55.8)  S: 50.6(47.2-54.0) | H: 22.1(19.2-25.1)  S: 20.4(17.6-23.2) | LiverAssist® device (XVIVO, Groningen, The Netherlands,  and Göteborg, Sweden) | 2.7(2.3-3.2) | H: 10.1(9.4-10.8)  S:10(9.2-10.7) | H: 58.1(52.0-64.2)  S: 52.4(46.4-58.5) | Pre-transplant graft preconditioning with HOPE results in higher hemodynamic stability during reperfusion and lower incidence of post-reperfusion syndrome and EAD. HOPE has the potential to mitigate electrolyte shifts by preventing hyperpotassemic complications that need to be addressed in liver transplantation with HOPE-pre-treated grafts |
| Patrono, 2022 | Italy | RCS | H: 121  S: 723 | H: 76.1(63.2-82.7)  S: 65.4(52.4-74.8) | H: 60.6(55.7-65.0)  S: 57.2(51.5-62.0) | H: 13.0(9.0-17.0)  S: 13.0(9.0-18.0) | Liver Assist device (XVivo, Groningen, The Netherlands) | 2.3(2.0-3.0) | H: 5.8(5.3-6.7)  S:7.3(6.4-8.2) | NA | HOPE for DBD grafts is associated with a lower rate of early allograft failure, postoperative complications, and reduced severity of ischemic cholangiopathy, resulting in improved patient and graft survival. |

CIT: cold ischemic time; DBD: donors after brain death; EAD: early allograft dysfunction; MELD: model for end-stage liver disease; PCS: prospective cohort study; RCS: retrospective cohort study; WIT: warm ischemic time.
